# Swainsonine Triggers Paraptosis *via* ER Stress and MAPK Signaling Pathway in Rat Primary Renal Tubular Epithelial Cells

**DOI:** 10.3389/fphar.2021.715285

**Published:** 2021-08-10

**Authors:** Shuai Wang, Yazhou Guo, Chen Yang, Ruijie Huang, Yuting Wen, Chunyan Zhang, Chenchen Wu, Baoyu Zhao

**Affiliations:** ^1^ College of Veterinary Medicine, Northwest A&F University, Yangling, China; ^2^ Institute of Poisonous Plants in Western China, Northwest A&F University, Yangling, China

**Keywords:** swainsonine, paraptosis, vacuolation, er stress, MAPK

## Abstract

Swainsonine (SW), an indolizidine alkaloid extracted from locoweeds, was shown toxic effects in multiple studies, but the underlying action mechanism remains unclear. SW is known to cause autophagy and apoptosis, but there has been no report on paraptosis mediated cell death. Here, we showed that SW induced rat primary renal tubular epithelial cells (RTECs) death accompanied by vacuolation *in vitro*. The fluorescence with the endoplasmic reticulum (ER)-Tracker Red and transmission electron microscopy (TEM) results indicated that the vacuoles were of ER origin, typical of paraptosis. The level of ER stress markers, such as polyubiquitinated proteins, Bip, CHOP and cytoplasmic concentration of Ca^2+^ have drastically increased. Interestingly, autophagy inhibitor could not interrupt but enhanced the induction of cytoplasmic vacuolization. Furthermore, MAPK pathways were activated by SW and inhibitors of ERK and JNK pathways could prevent the formation of cytoplasmic vacuolization. In this study, we confirmed that SW induced cell paraptosis through ER stress and MAPK signaling pathway, thus further laying a theoretical foundation for the study of SW toxicity mechanism.

## Introduction

Swainsonine (SW), an indolizidine alkaloid, is the principal toxic component of locoweeds (its name originated from Spanish “loco” meaning crazy), and is produced by fungi living within locoweeds (Cook et al., 2017). Currently, locoweed has been found throughout the world and has become the major toxic plant affecting the livestock production in pastureland (Lu et al., 2014). Locoweed poisoning causes huge economical loss annually which severely hampered the development of the grassland (Daniel et al., 2009; Wei et al., 2015; Pfister et al., 2016).

The research shows that swainsonine can cause injury of multiple tissues/organs of grazing livestock, and the extensive vacuolar degeneration is major pathological manifestation. However, the underlying mechanisms involved in SW-induced animal poisoning remain poorly understood. Recent studies indicate that SW induces a variety of cell apoptosis, such as rat cardiomyocytes Zheng et al. (2018), cerebral cortical neurons Lu et al. (2015), and caprine luteal cells (Li et al., 2015). In addition, our earlier study also showed that SW can activate autophagy in rat primary renal tubular epithelial cells (RTECs) (Wang et al., 2019). Although it has been confirmed that SW can cause the accumulation of complex carbohydrates and glycoproteins in cells due to its toxic effects, thereby inducing apoptosis and autophagy, there may be other toxic mechanisms.

Paraptosis is a type of programmed cell death displaying cytoplasmic vacuolation, usually consisting in mitochondrial and/or ER swelling. It was first described by (Sperandio et al., 2000). Previous studies show that paraptosis requires protein synthesis and can be blocked by the translation inhibitor cycloheximide (CHX)(Sperandio et al., 2004). Unlike apoptosis, paraptosis does not require activation of caspases or formation of apoptotic bodies (Fontana et al., 2020).

Although the mechanisms of paraptosis, particularly the mechanisms responsible for triggering dilation of mitochondria or the ER, have not yet been entirely clear, paraptosis is usually accompanied by an alteration of Ca^2+^ Yoon et al. (2012) and redox homeostasis Yoon et al. (2010), as well as by proteostasis disruption Seo et al. (2019) and ER stress (Fabrizio et al., 2019; Zhang et al., 2020; Nedungadi et al., 2021). Ultimately, unfolded/misfolded proteins accumulate in the ER lumen, leading to the activation of pro-death processes. However, these features are not always present in cells undergoing paraptosis (Fontana et al., 2020).

In this study, we investigated the possible involvement of non-canonical programmed cell deaths (i.e. paraptosis) in the toxic effects of SW. Our data revealed that paraptosis caused by SW was a cell death pathway different from apoptotic and autophagic. In summary, we report for the first time that SW can inhibit proteasome function and induce ER stress, leading to ER dilation and the subsequent cell paraptosis. In addition, JNK and ERK pathways play an important role in the cytoplasmic vacuolization induced by SW.

## Materials and Methods

### Materials

**FIGURE 1:**
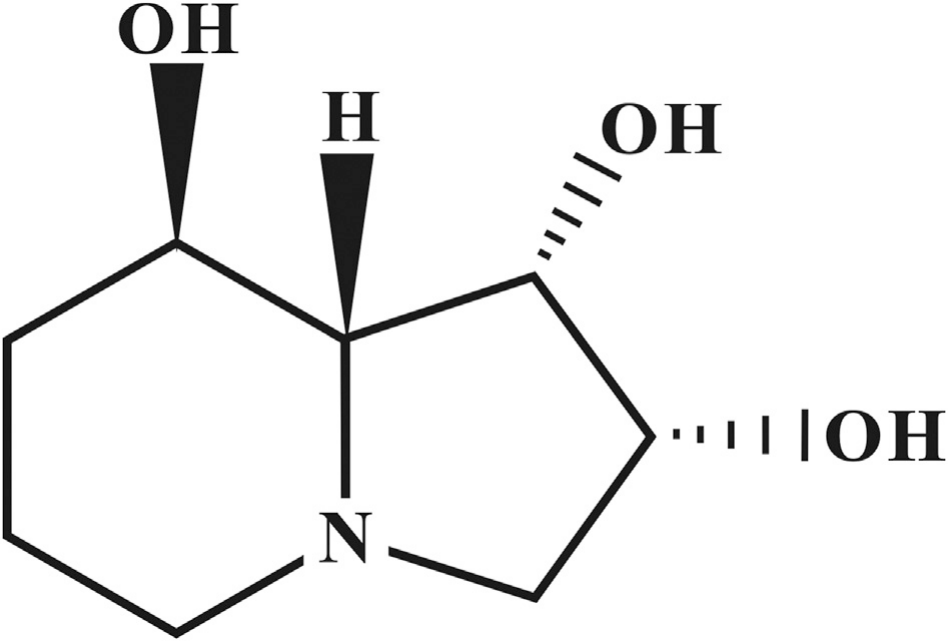
The chemical structure of swainsonine.

SW (Figure 1) was isolated from *Oxytropis kansuensis* Bunge (a locoweed widely distributed in western China) and identified by interpretation of spectral data (MS, 1H NMR, 13C NMR, 2D NMR) as described previously (Lu et al., 2012). Its purity was 98.17%. Bafilomycin A1 (Baf A1) (HY-100558), Rapamycin (Rapa) (HY-10219), U0126 (HY-12031), SP600125 (HY-12041) and 4-Phenylbutyric acid (4-PBA) (HY-A0281) were obtained from MCE, United States p-PERK (#3179, Rabbit, anti-rat), ATF6 (#65880, Rabbit, anti-rat), p38 (#8690, Rabbit, anti-rat), and p-p38 (#4092, Rabbit, anti-rat) antibodies were purchased from Cell Signaling Technology, United States. Caspase Inhibitor z-VAD-fmk, CHX, ER-Tracker Red, Fluo-4 AM, eIF2α(AF6771, Rabbit, anti-rat) and p-eIF2α(AF1237, Rabbit, anti-rat) were purchased from Beyotime (Shanghai, China). Bip (CY5166, Rabbit, anti-rat), Alix (CY7215, Rabbit, anti-rat), p-IRE1 (CY5605, Rabbit, anti-rat), JNK(CY5623, Rabbit, anti-rat), p-JNK (CY5541, Rabbit, anti-rat), Ubiquitin (CY5520, Rabbit, anti-rat) and β-actin (AB0035, Rabbit, anti-rat) antibodies were purchased from Abways (Shanghai, China). PERK (A01992-2, Rabbit, anti-rat), CHOP (BM4962, Rabbit, anti-rat), IRE1 (A00683-1, Rabbit, anti-rat), ERK (BM4326, Rabbit, anti-rat), p-ERK (BM4156, Rabbit, anti-rat) and Goat Anti-Rabbit IgG (BA1054) was purchased from BOSTER (Wuhan, China). Live and Dead^TM^ Viability/Cytotoxicity Assay Kit was purchased from US Everbright® Inc. (Suzhou, China).

### Cell Culture and Drug Treatment

Renal tubular epithelial cells (RTECs) were prepared as previously described Rudolfs and Lawrence (1996), Liu et al. (2016) from SD rats. RTECs were maintained in DMEM (Gibco) supplemented with 10% fetal bovine serum (FBS) (Gibco) and 100 U/mL penicillin/streptomycin (Sigma) at 37 C and 5% CO_2_.

The cells were cultured in a medium with SW (0–400 μg/ml) for 24 h or retreated with Baf A1 (10 nM), Rapa (50 nM), z-VAD-fmk (2 μM), 4-PBA (1 mM), SP600125(20 μM), U0126 (15 μM) or CHX (2 μM) for 2–4 h and then cultured in medium for 24 h. After reaching 70–80% confluence, the cells were harvested for the subsequent biochemistry analysis.

### Measurement of Cellular Viability

For measurement of cellular viability, cells were cultured in 12-well plates and treated with SW. According to the instructions of Live and Dead^TM^ Viability/Cytotoxicity Assay Kit, 2 μM calcein-acetoxymethyl ester (calcein-AM), a green fluorescent indicator of the intracellular esterase activity of cells, and 4 μM propidium (PI), a red fluorescent indicator of membrane damaged/dead cells, we added to each well, and the plates were incubated for 30 min in 5% CO_2_ at 37°C. The calcein-positive live cells and PI-positive dead cells were visualized using a fluorescence microscope (OLYMPUS-IX71, Japan) and counted.

### ER Localization With ER-Tracker

ER staining was performed according to the instructions of ER-Tracker Red kit. After treatment, RTECs were washed twice with PBS and then incubated in pre-warmed ER-tracker dye solution (1 mM) for approximately 30 min at 37°C. The cells were then observed using a fluorescence microscope (OLYMPUS-IX71, Japan).

### Analysis of Ca^2+^ Accumulation Using Fluoresce Microscopy

Fluo-4 AM is cleaved into Fluo-4 by esterases in cells and Fluo-4 combines with Ca^2+^ to produce stronger fluorescence. To measure cytosolic Ca^2+^ levels, cells were grown on 6-well plates and treated with SW. Then, Fluo-4 AM was added to the medium at a final concentration of 1 µM. After a 30 min incubation at 37°C, cells were washed with PBS, and visualized by fluorescence microscopy (OLYMPUS-IX71, Japan).

### Western Blotting

After treatment, RTECs were harvested and washed with ice-cold PBS. Total protein was then extracted from the cells using ice-cold RIPA lysis buffer (Solarbio, Beijing, China) containing 1 mM PMSF. Protein concentrations were calculated using BCA assay kits (Solarbio, Beijing, China), and 20 μg of protein was subjected to 12% SDS-PAGE and transferred to PVDF membranes (Millipore, United States). The membranes were blocked with 5% nonfat milk powder at room temperature for 2 h, and immunoblotting was performed with primary antibodies at 4°C overnight, followed by HRP-conjugated secondary antibody at room temperature for 2 h. Following each step, the membranes were washed five times with TBS-T for 5 min. All antibodies (including Bip, CHOP, Alix, PERK, IRE1, and JNK etc.) were diluted at 1:5,000, according to the protocols. The proteins were visualized using enhanced chemiluminescence (Peiqing, JS-1070EV, China).

### Transmission Electron Microscopy (TEM) Observation

*In vitro* experiments, after treatment, RTECs were washed three times with PBS, and fixed with 4% glutaraldehyde and postfixed with 1% O_s_O_4_ in 0.1 M cacodylate buffer containing 0.1% CaCl_2_ for 2 h at 4°C. The samples were then stained with 1% Millipore-filtered uranyl acetate, dehydrated in increasing concentrations of ethanol, infiltrated, and embedded. After polymerization of the resin at 60°C for 48 h, ultrathin sections were cut with an ultracut microtome. Sections were stained with 4% uranyl acetate and lead citrate, and images were obtained using a transmission electron microscope (HITACHI, HT7800, Japan).

### Statistical Analysis

All results were expressed as the mean ± standard deviation (S.D.). Data were obtained from three independent experiments. All statistical analyses were conducted by GraphPad Prism 5 software (San Diego, CA). Data were analyzed using paired t-tests. Significant differences: * equals *p* < 0.05; ** equals *p* < 0.01.

## Results

### SW Induced Cytoplasmic Vacuolization and Cell Death in RTECs

**FIGURE 2:**
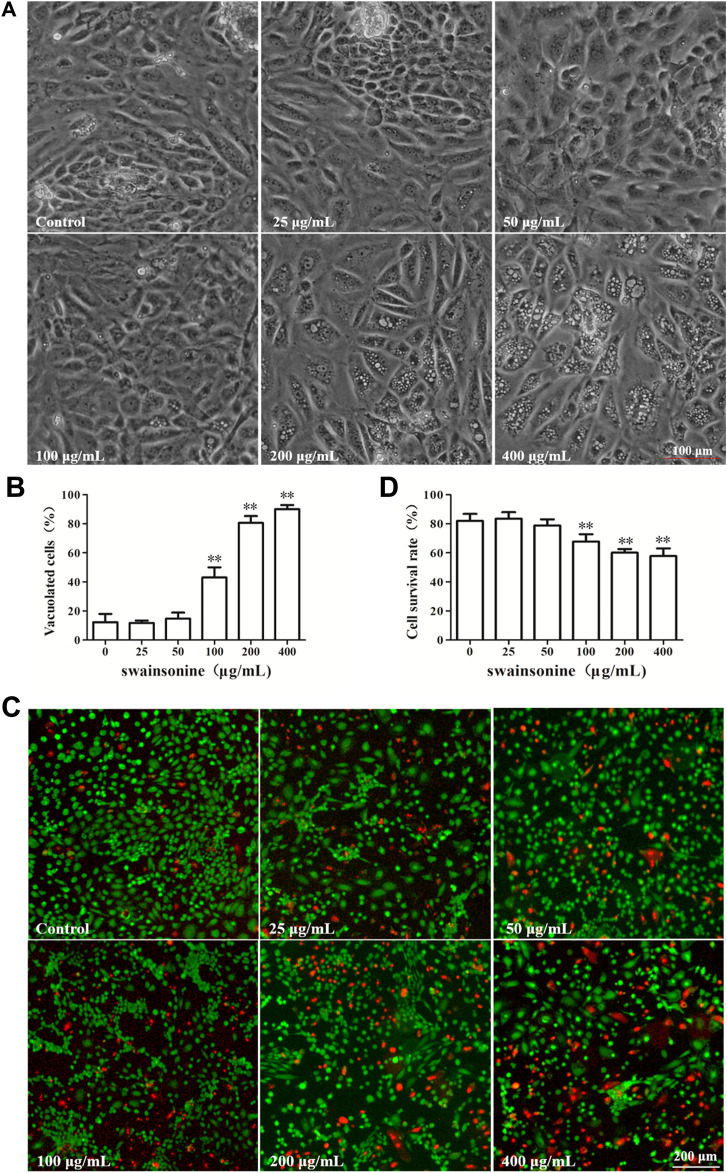
SW induced cytoplasmic vacuolization and inhibited cell viability in RTECs **(A,B)** Phase-contrast images of RTECs following incubation with SW at the indicated concentrations for 24 h. Morphometric analysis of vacuoles was performed with three different areas and the percentage of SW-induced vacuolated cells was calculated **(C,D)** RTECs were treated with the indicated concentrations of SW for 24 h and cellular viability was measured using Live and Dead^TM^ Viability/Cytotoxicity Assay Kit. Analyze the number of living (green spots) and dead cells (red spots) in three different areas of control and SW-treated cells and the ratios were calculated.

The cytotoxic effects of SW was evaluated in RTECs. The RTECs were cultured with various doses of SW (0, 25, 50, 100, 200, and 400 μg/ml) for 24 h. Microscopic observation showed that SW treatment induced extensive cytoplasmic vacuolation in RTECs. A significantly higher quantity of cytoplasmic vacuolated cells was observed when cells were treated with 400 μg/ml SW compared to that of control cells or cells that were treated with lower doses of SW (Figure 2A, B).

After 24 h treatment, some of the cells became detached from the culture plate with intracellular vacuole surrounding the cell nucleus. To detect the effect of SW on cell survival rate, the RTECs were then treated with SW (0, 25, 50, 100, 200 and 400 μg/ml) for 24 h, and cell viability was then determined using Live and Dead^TM^ Viability/Cytotoxicity Assay Kit. We found that exposure to SW obviously decreased the viability of RTECs in a dose-dependent manner (Figures 2C, D), suggesting that SW has cytotoxic activity against RTECs.

### SW-Induced Cytoplasmic Vacuolation Is Independent of Apoptosis and Autophagy

We have previously confirmed that SW can induce apoptosis and autophagy, but which cell death pathway is related to SW-induced cytoplasmic vacuolation is still unclear.

**FIGURE 3:**
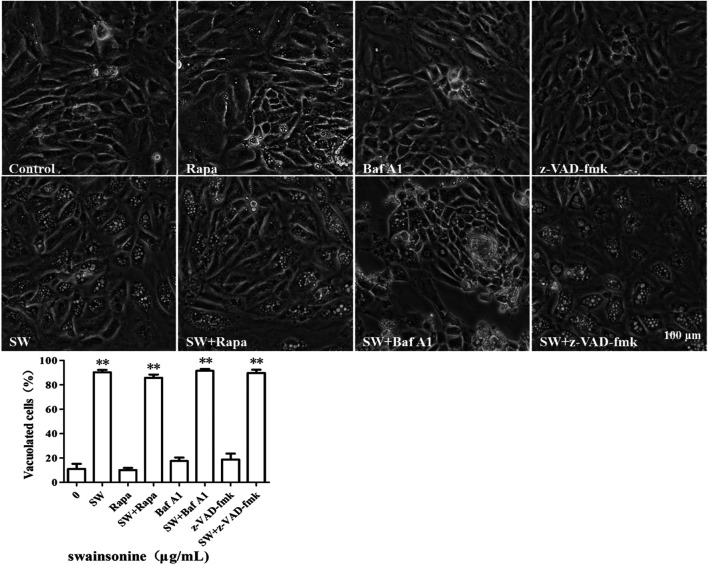
SW induced cytoplasmic vacuolization which is independent of autophagy and apopotosis. Inverted microscopic images of RTECs treated with SW (400 μg/ml) in the presence of autophagic activators like Rapa, autophagic inhibitors like Baf A1, and apoptosis inhibitors like z-VAD-fmk for 24 h. Morphometric analysis of vacuoles was performed with three different areas and the percentage of SW-induced vacuolated cells was calculated.

We pre-treated the cells with autophagic inhibitor like Baf A1 and autophagy activator like Rapa and found that the treated cells continued to vacuolate even in the presence of the inhibitor or activator (Figure 3). It is noteworthy that the combination of both SW and autophagic inhibitor (Baf A1) lead to increase in cell death when compared to death by SW alone (Supplementary Figure S1) suggesting that the blockage of autophagy resulted in increased mortality rate of cells treated with SW.

To further assess the possible involvement of apoptosis, we pre-treated the cells with the pan-caspase inhibitor, z-VAD-fmk. However, we found that pretreatment with z-VAD-fmk did not affect the vacuolation or cell death of SW-treated cells (Figure 3 and Supplementary Figures S1, S2). Collectively, these results showed that an alternative cell death mode might be involved in the cytotoxic effects induced by SW.

### SW Caused ER Stress-dependent Paraptosis of RTECs

Cytoplasmic vacuolation arising from ER dilation and/or swelling of mitochondria is one of the major hallmarks of paraptosis (Yoon et al., 2012; Bury et al., 2013). To further evaluate the cell death mode induced by SW in RTECs, we examined the origins of the SW-induced vacuoles.

**FIGURE 4:**
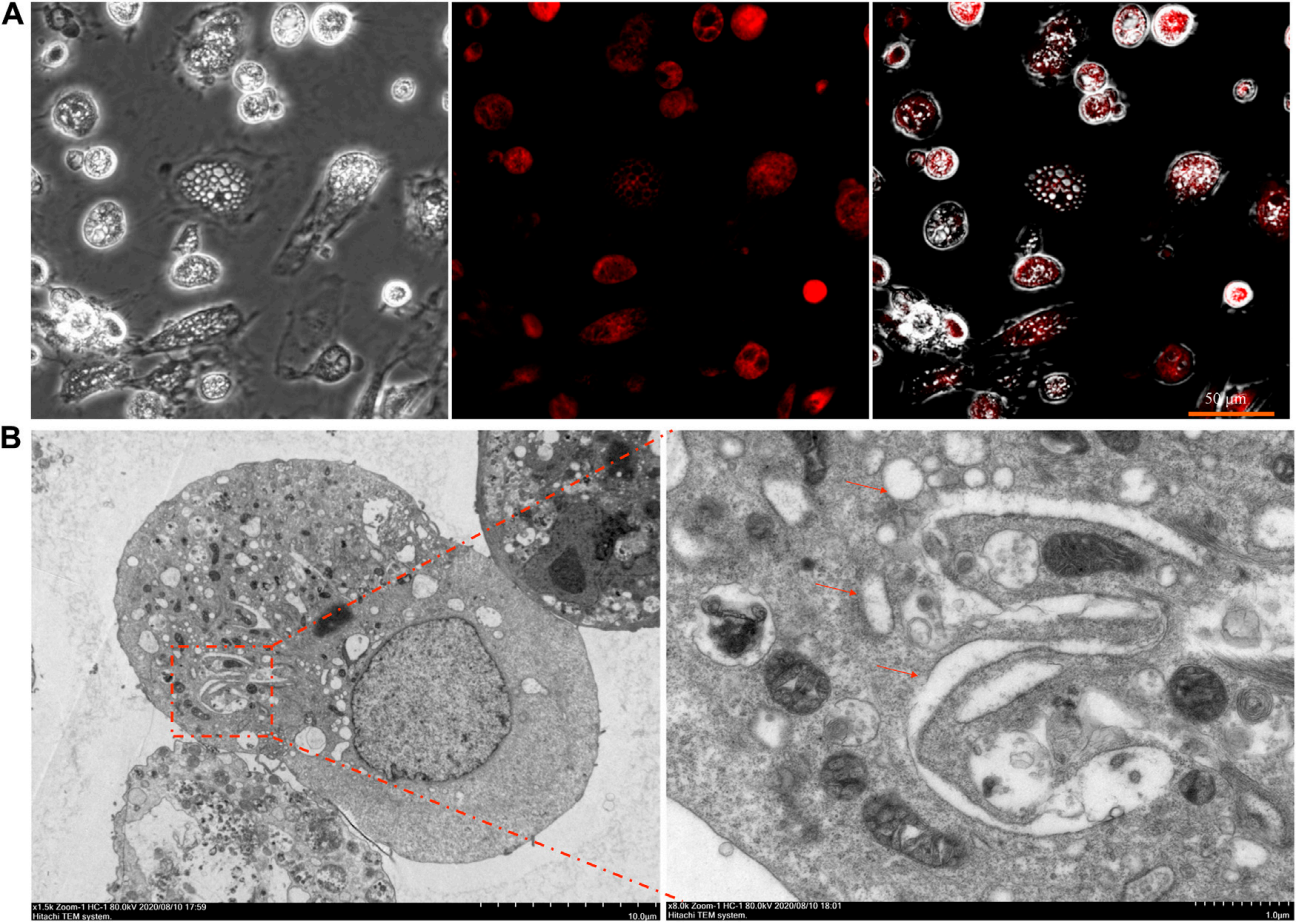
Fluorescence staining and ultrastructure of SW-induced cytoplasmic vacuolation **(A)** The cytoplasmic vacuolation induced by SW in RTECs was observed by fluorescence staining with ER-tracker **(B)** RTEC cells were treated with SW for 24 h and electron microscopy was performed. Arrows indicate the dilated ER.

The fluorescence microscopy of cells loaded with the ER tracker Red showed that the membranes of large vacuoles were stained with the ER tracker (Figure 4A). In addition, it is seen from the TEM data that, during the incubation of cells in the presence of SW, mitochondria almost do not change in size and are not colocalized with vacuoles. However, the TEM images with 24 h of treatment with SW clearly depicts large empty vacuoles appeared to be very close to ER (Figure 4B), stating that the cytoplasmic vacuoles originated from endoplasmic reticulum.

**FIGURE 5:**
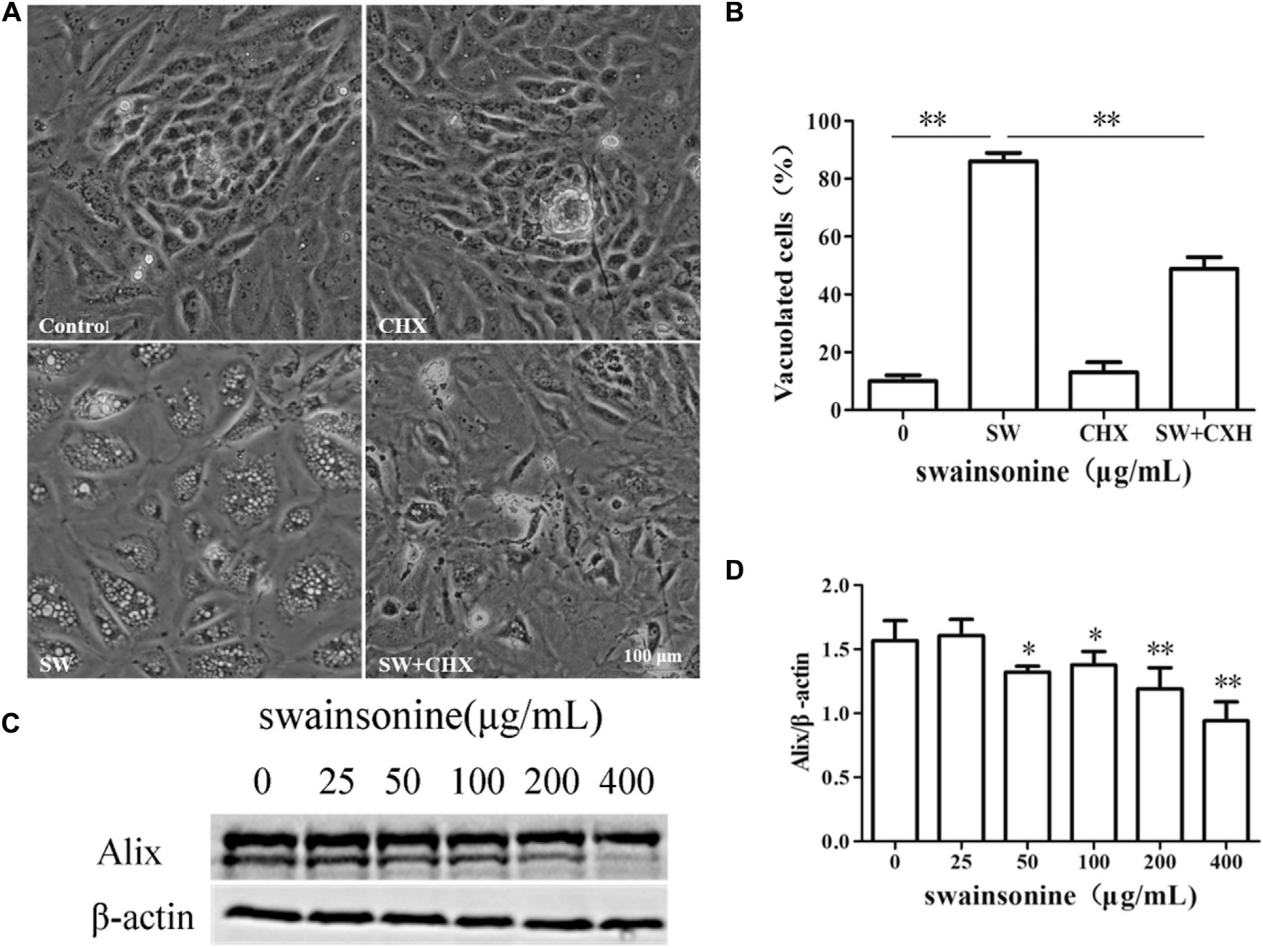
SW induced paraptosis in RTECs **(A,B)** RTECs were pretreated with CHX for 4 h and further treated with 400 μg/ml SW for 24 h. Inverted microscopic images of RTECs treated with SW (400 μg/ml) in the presence of CHX. Morphometric analysis of vacuoles was performed with three different areas and the percentage of SW-induced vacuolated cells was calculated **(C,D)** Alix expression level was evaluated by Western blot analysis in RTECs treated with SW at the indicated concentrations for 24 h. Three independent experiments were performed. Statistically significance was indicated: *p < 0.05, **p < 0.01 compared with control.

We next examined whether SW-induced cell death in RTECs shared other features of paraptosis. Although the molecular basis of paraptosis still remains to be clarified, it is known to require *de novo* protein synthesis. Accordingly, we tested the effect of pretreatment with the protein synthesis blocker, CHX, and found that it very effectively blocked SW-induced vacuolation in RTECs (Figure 5A+B). However, the co-treatment of SW and CHX aggravated cell death (Supplementary Figure S3), which may be due to the hindrance of protein synthesis aggravating the toxic effects of SW. In addition, the levels of Alix, an inhibitor of paraptosis, were downregulated by SW in RTECs (Figure 5C, D). These results indicated that SW induced paraptosis in RTECs.

**FIGURE 6:**
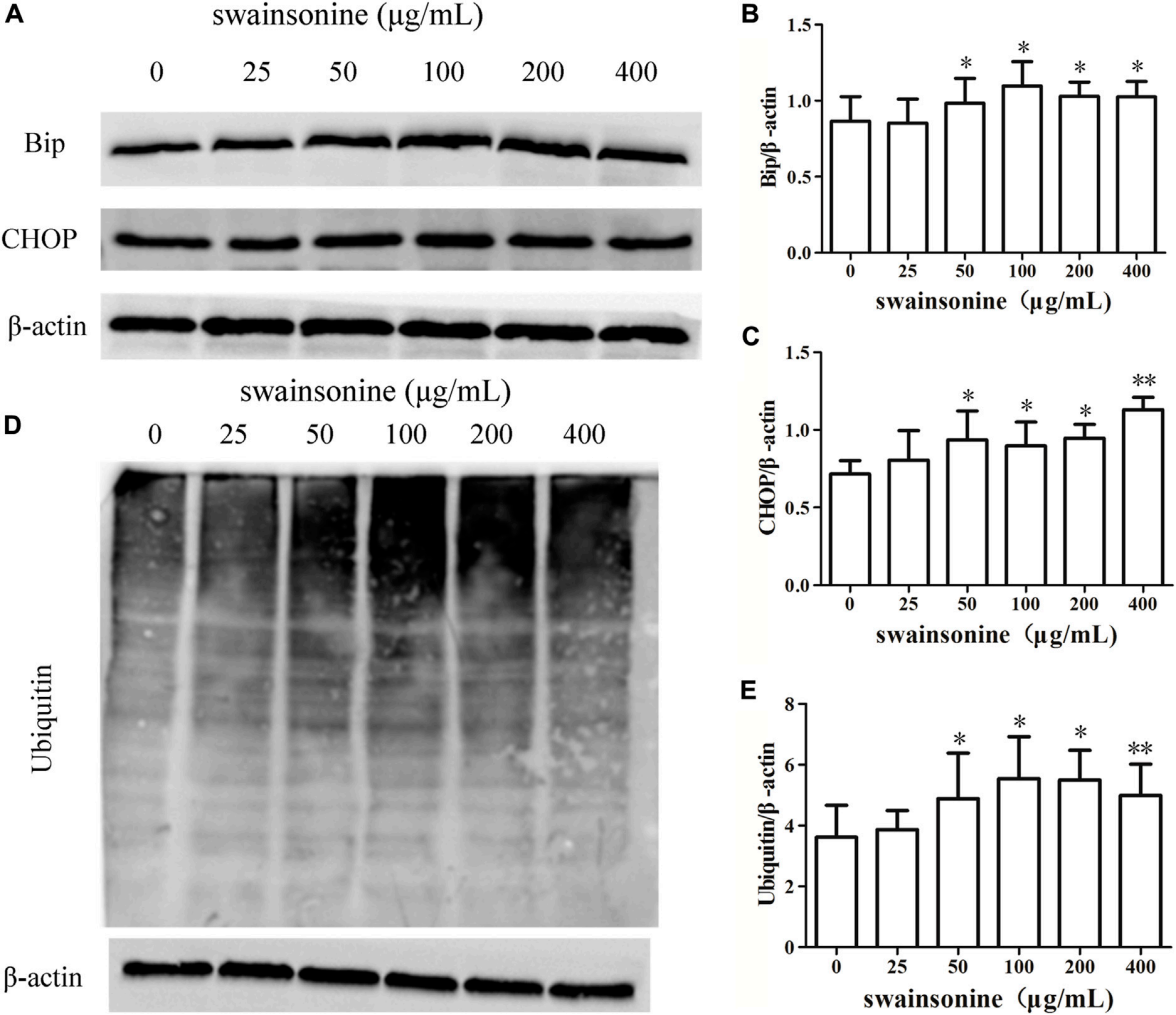
SW increases the expression of proteins involved in ER stress in RTECs. RTECs were exposed to 0–400 μg/ml SW for 24 h, and western blotting showing the expression of Bip, CHOP **(A–C)** and polyubiquitinated proteins **(D + E)** in cells. Three independent experiments were performed. Statistically significance was indicated: *p < 0.05, **p < 0.01 compared with control.

The dilation of ER suggested the presence of ER stress in RTECs with SW treatment, and paraptosis mediated cell death is often associated with ER stress (Lee et al., 2016). Furthermore, proteasome inhibition has been shown to induce the accumulation of misfolded proteins in the ER lumen and to impose ER stress (Nawrocki, 2005). Recent studies have shown that the occurrence of apoptosis is related to proteasome inhibition. Thus we checked for the protein expression levels of the ER stress markers like Bip and Chop in RTECs following treatment with SW (Tabas and Ron, 2011; Tang et al., 2012). We found that there was a notable increase in the expression of these markers in a dose dependent manner (Figures 6A–C). Western blot analysis using an anti-ubiquitin antibody demonstrated progressive accumulation of polyubiquitinated proteins also in SW-treated cells (Figure 6D, E).

**FIGURE 7:**
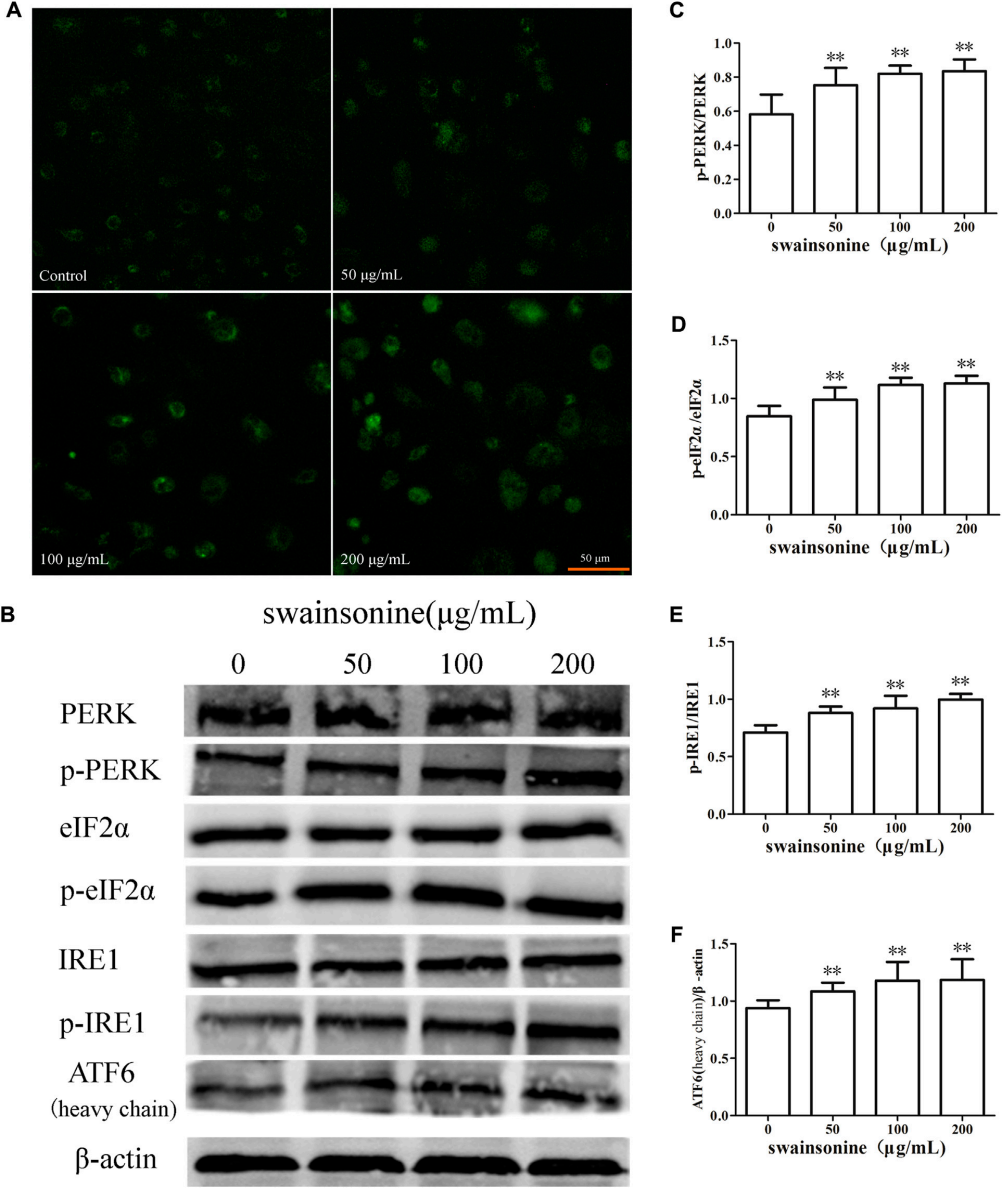
SW increases Ca^2+^ concentration and activates pathways related to ER stress in RTECs. RTECs treated with various doses of SW for 24 h **(A)** SW treatment caused an increase in Ca^2+^ concentrations in RTECs compared with untreated cells **(B)** PERK, eIF2α, IRE1, ATF6 expression levels were evaluated by Western blot analysis **(C–F)** These column charts show the ratio of p-PERK/PERK, p-eIF2α/eIF2α, p-IRE1/IRE1, ATF6/β-actin. Three independent experiments were performed. Statistically significance was indicated: *p < 0.05, **p < 0.01 compared with control.

It is known that one of the factors inducing the ER stress is the disturbance of the homeostasis of intracellular Ca^2+^ (Carreras-Sureda et al., 2017). Ca^2+^ is released into the cytoplasm when the ER structure is abnormal. As shown in Figure 7A, stronger fluorescence intensity was observed in SW-treated cells, indicating that SW treatment increased intracellular Ca^2+^ concentration.

Also, we determined the changes in protein expression of three other ER transmembrane sensors, IRE1, PERK and ATF6 Wang and Kaufman (2014), and our results showed that the expression of p-PERK, p-IRE1, and ATF6 increased in RTECs when the cells were treated with SW (Figures 7B–F).

**FIGURE 8:**
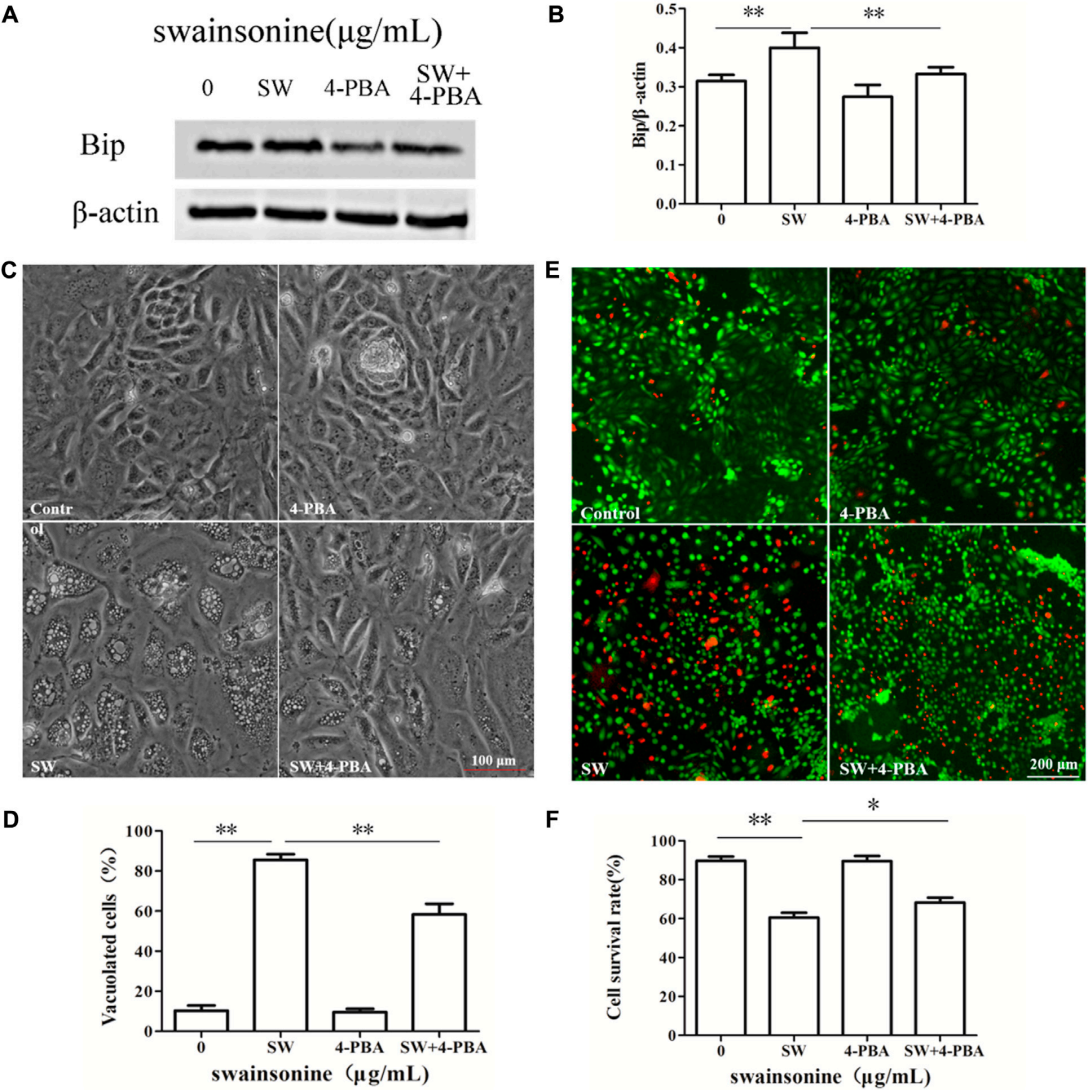
Effects of 4-PBA on SW-induced cytoplasmic vacuolization and cell death. RTECs were pretreated with 4-PBA at the indicated concentrations for 4 h and further treated with 400 μg/ml SW for 24 h **(A,B)** Western blotting was used to analyze the Bip protein level changes in the presence of 4-PBA. Three independent experiments were performed. *p < 0.05, **p < 0.01 **(C,D)** Inverted microscopic images of RTECs treated with SW (400 μg/ml) in the presence of 4-PBA. Morphometric analysis of vacuoles was performed with three different areas and the percentage of SW-induced vacuolated cells was calculated **(E,F)** Cellular viability was assessed using Live and Dead^TM^ Viability/Cytotoxicity Assay Kit. Analyze the number of living (green spots) and dead cells (red spots) in three different areas of control and SW-treated cells and the ratios were calculated.

It is worth noting that pre-treatment of RTECs with the ER stress inhibitor 4-PBA markedly suppressed SW-induced cytoplasmic vacuolation and cell death (Figure 8), supporting the relationship between vacuoles and ER stress. In conclusion, ER stress plays an important role in SW induced paraptosis.

### MAPK Activation Mediates Paraptosis Induced by SW

According to existing knowledge, the MAPK signal transduction pathways have been identified to be involved in the process of paraptosis induction (Wang et al., 2012; Li et al., 2020). To understand the detailed mechanism by which SW treatment induces paraptosis in RTECs, we analyzed the signal activation of the p38/Erk/JNK MAPK pathway in cells treated with SW.

**FIGURE 9:**
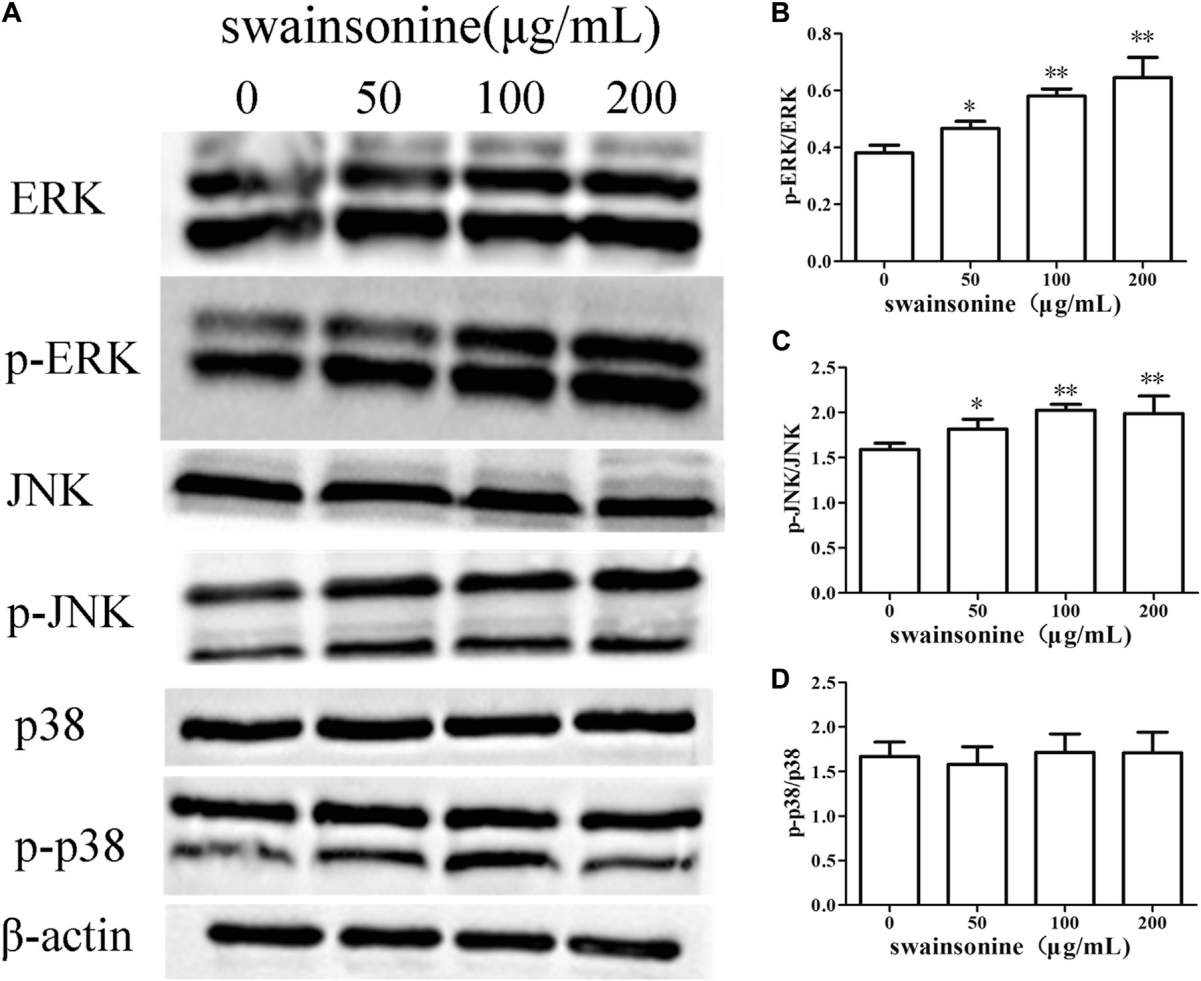
SW increases the expression of proteins involved in the MAPK cascade in RTECs **(A)** The proteins levels changes of ERK, JNK and p38 were analyzed by the Western blotting analysis of cells treated with a range of concentration of the SW for 24 h **(B–D)** These column charts show the ratio of p-ERK/ERK, p-JNK/JNK, p-p38/p38. Three independent experiments were performed. Statistically significance was indicated: *p < 0.05, **p < 0.01 compared with control.

Western blot analysis revealed that treatment with SW upregulated the phosphorylation levels of ERK and JNK in RTECs compared to those of the control cells. At the same time, SW did not cause significant change in total levels of ERK and JNK. However, p38 activity was not affected after treatment of RTECs with SW (Figure 9).

**FIGURE 10:**
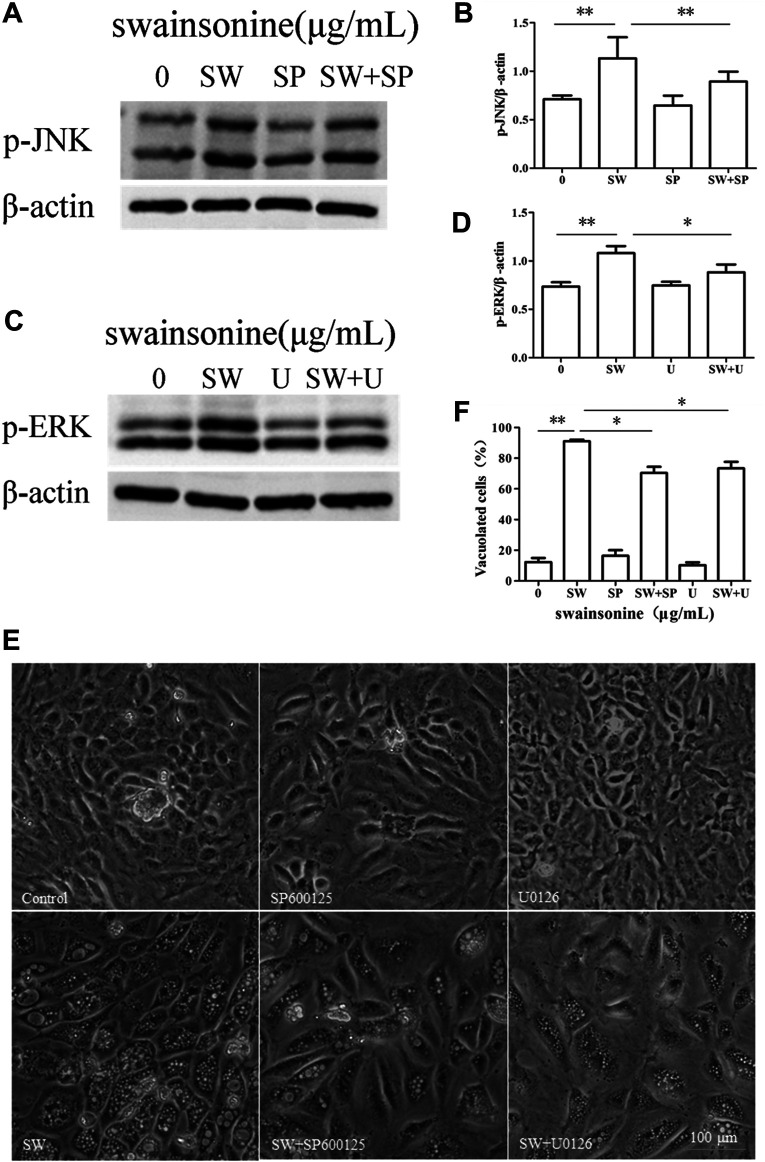
Effects of MAPK pathway inhibitors on SW-induced cytoplasmic vacuolization. RTECs were pretreated with SP600125 or U0126 at the indicated concentrations for 4 h and further treated with 400 μg/ml SW for 24 h **(A–D)** Western blotting was used to analyze the *p*-JNK and *p*-ERK protein level changes. Three independent experiments were performed. *p < 0.05, **p < 0.01 **(E,F)** Inverted microscopic images of RTECs treated with SW (400 μg/ml) in the presence of SP600125 or U0126. Morphometric analysis of vacuoles was performed with three different areas and the percentage of SW-induced vacuolated cells was calculated.

Then we used specific inhibitors of MAPK pathways to check the effect of MAPK inhibition on cytoplasmic vacuolization induced by SW. Pretreatment of RTECs with either U0126, a ERK inhibitor, or SP600125, a JNK inhibitor, partially but significantly attenuated SW-induced vacuolation (Figure 10), but not markedly reduce cell death (Supplementary Figure S4). These results indicated that ERK and JNK pathways were involved in the cytoplasmic vacuolization induced by SW.

## Discussion

SW is a kind of indolizidine alkaloid, which was first isolated from the plant *Swainsona canescens* (Dawn et al., 1984; Gardner et al., 2001; Colodel et al., 2002; Haraguchi et al., 2003). Consumption of SW-containing locoweeds by livestock can cause animal poisoning, and the extensive vacuolar degeneration is major pathological manifestation. It is known that SW can induce apoptosis and autophagy. However, a drug can cause more than one kind of programmed cell death at the same time. For example, Iturin A-like lipopeptides induces paraptosis, accompanied by autophagy and apoptosis in cancer cells (Zhao et al., 2018).

Paraptosis is a kind of recently defined programmed cell death which differ from the classical apoptosis by lacking caspase activation, the formation of apoptotic bodies. The presence of cytoplasmic vacuolation arising either from swelling of mitochondria or ER dilation a significant feature of paraptosis. Although the mechanism of paraptosis still remains to be clarified, it is known to be associated with ER stress, disturbances in the Ca^2+^ distribution in cells Yoon et al. (2014b), Dong et al. (2017) and the perturbation of cellular proteostasis via proteasomal inhibition (Yoon et al., 2010; Yoon et al., 2014a; Mnich et al., 2015; Dongjoo et al., 2016). Also, evidence shows that activation of MAPK pathway and disruption of sulfur homeostasis may lead to the induction of paraptosis (Yoon et al., 2010; Chen et al., 2018; Dongrong et al., 2019).

Here we reported that SW was able to induce extensive cytoplasmic vacuolation mediated cell death in RTECs. In order to investigate whether SW-induced vacuolar degeneration is related to autophagy and apoptosis, we used Rapa (an autophagy activator), Baf A1 (a late-phase autophagy inhibitor) and z-VAD-fmk (an apoptosis inhibitor). However, the tested reagents did not significantly affect the SW-induced cell death and vacuolation of RTECs. Furthermore, autophagy inhibitors did not prevent but, on the contrary, enhanced the formation of cytoplasmic vacuolization induced by SW, suggesting that cytoplasmic vacuolation was due to other cell death modes.

To check whether the cytoplasmic vacuolization was resulted from paraptosis, we examined its relationship with ER structure. The results of fluorescence staining and transmission electron microscopy showed that the vacuoles originated from ER. Incubation of cells with the protein synthesis inhibitor CHX decreased cytoplasm vacuolization. In addition, SW reduced the expression of Alix in RTECs. These results suggested that the cytoplasmic vacuolization induced by SW was related to paraptosis.

We next discussed the mechanism of SW-induced paraptosis. When ER stress occurs, the expression of Bip increases and three stress receptors (PERK, IRE1 and ATF6) are activated. And, the transcription factor CHOP plays a key role in the ER stress-related apoptosis pathway (He and Klionsky, 2009; Mnich et al., 2015). We found an increase in ER stress markers like Bip, CHOP, p-PERK, p-IRE1 and ATF6 at protein level in SW treated RTECs.

ER is considered to be the major store house of Ca^2+^ and Ca^2+^ is a unique secondary messenger which plays an important role in the proper folding of proteins in the cells. One of the factors known to cause ER stress is the disorder of intracellular Ca^2+^ homeostasis. We observed an increase in the cytosolic concentration of Ca^2+^ in SW treated cells which further indicates the presence of ER stress.

In addition, we also checked the effect of SW on the proteasome in RTECs. The ubiquitin proteasome system (UPS) is a central component of the cellular protein degradation machinery. It can prevent the accumulation of misfolded or deleterious proteins in the cell and performs essential functions in homeostasis. Many newly synthesized error proteins are degraded by proteasome (Schubert et al., 2000). SW treatment induced accumulation of ubiquitinated protein which indicated that it had inhibitive effect on proteasome function.

Previously, Mimnaugh (2006) proposed that a proteasome-inhibition-triggered overload of misfolded proteins in the ER lumen could exert an osmotic force that draws water from the cytoplasm and induced extensive ER-derived vacuolization. And, the application of the ER stress inhibitor 4-PBA, which affects the protein folding and traffic Zhang et al. (2013), Kolb et al. (2015), alleviated SW-induced vacuolation and slightly inhibited the cytotoxic effect. Thus, we believe that ER stress has a key role in SW-induced cytotoxicity.

In our research, though SW activated MAPK pathways, including p38, ERK and JNK, paraptosis induced by SW was only dependent on JNK and ERK pathways. Because our results showed that U0126 and SP600125 reduced the number of vacuolated cells underwent SW treatment.

**FIGURE 11:**
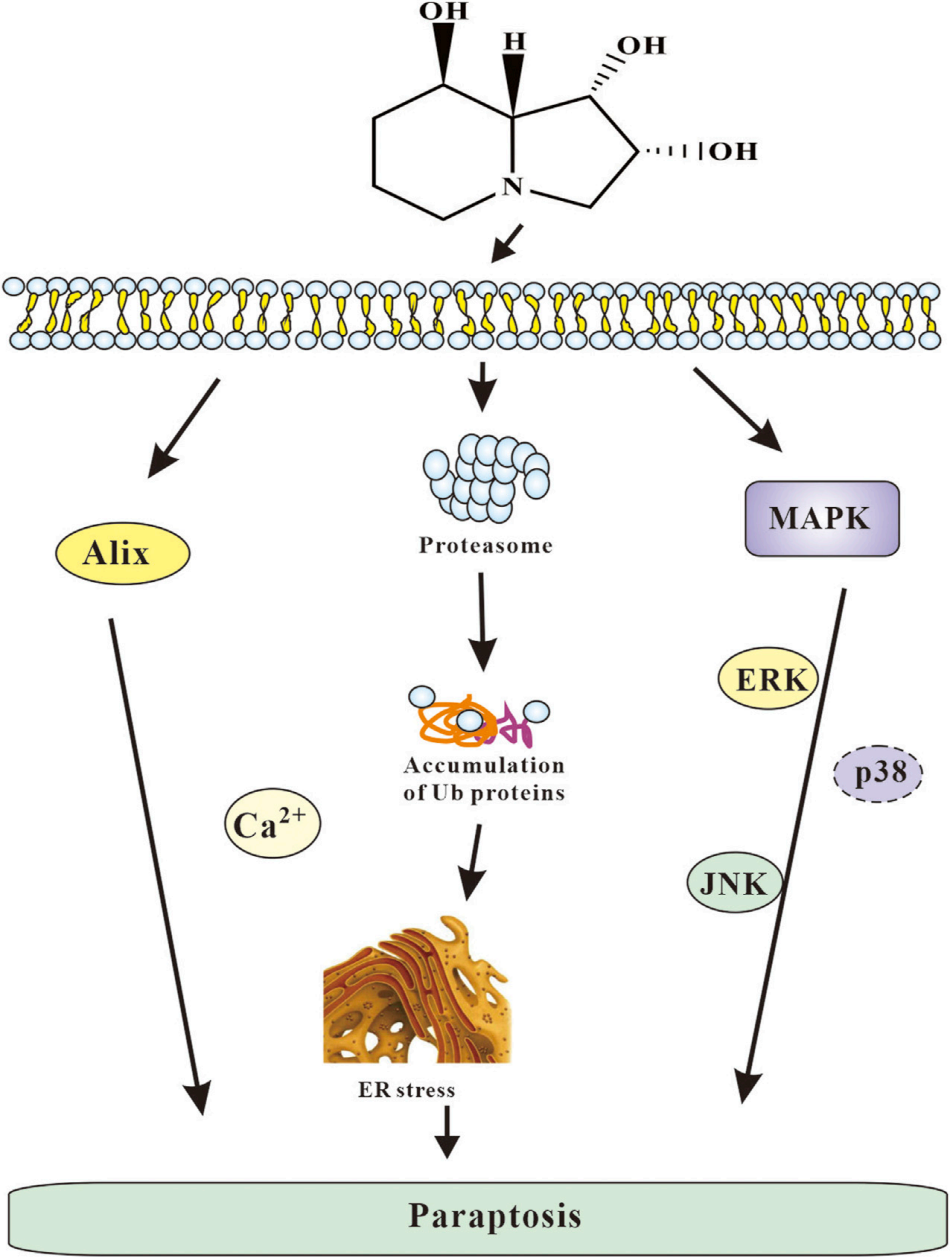
The overall mechanism of SW induced cell paraptosis.

In summary, paraptosis induced by SW contributed to cytotoxicity of SW might be based on its capability to activate several pathways such as proteasome inhibition, ER stress and MAPK passway. Based on the results obtained, the following mechanism of the cytotoxic action of SW can be proposed (Figure 11): SW cause ER stress, an increase in the intracellular Ca^2+^ concentration, and damage to proteasomal. Consequently, the accumulation of misfolded proteins in ER results in a drastic extension of the ER cisterns and an extensive cytoplasmic vacuolization, the disturbance of the allocation of organelles by giant vacuoles, which leads to the initiation of cell death caused by paraptosis. Moreover, JNK and ERK pathways play an important role in this mode of cell death.

## Data Availability

The original contributions presented in the study are included in the article/Supplementary Material, further inquiries can be directed to the corresponding authors.

## References

[B1] Bury M. Girault A. Mégalizzi V. Spiegl-Kreinecker S. Mathieu V. Berger W., (2013). Ophiobolin A Induces Paraptosis-like Cell Death in Human Glioblastoma Cells by Decreasing BKCa Channel Activity. Cel Death Dis. 4, e56. 10.1038/cddis.2013.85 PMC3615734 23538442

[B2] Carreras-Sureda A. Pihán P. Hetz C. (2017). Calcium Signaling at the Endoplasmic Reticulum: fine-tuning Stress Responses. Cell Calcium 70, 20–31. 10.1016/j.ceca.2017.08.004 29054537

[B3] Chen X. Chen X. Zhang X. Wang L. Cao P. Rajamanickam V. (2018). Curcuminoid B63 Induces ROS-Mediated Paraptosis-like Cell Death by Targeting TrxR1 in Gastric Cells. Redox Biol. 21, 101061. 10.1016/j.redox.2018.11.019 30590310 PMC6306695

[B4] Colodel E. M. Gardner D. R. Zlotowski P. Driemeier D. (2002). Identification of Swainsonine as a Glycoside Inhibitor Responsible for Sida Carpinifolia Poisoning. Vet. Hum. Toxicol. 44, 177–178. 12046976

[B5] Cook D. Donzelli B. G. G. Creamer R. Baucom D. L. Gardner D. R. Pan J. (2017). Swainsonine Biosynthesis Genes in Diverse Symbiotic and Pathogenic Fungi. G3 & 58 Genesgenetics 7, 1791–1797. 10.1534/g3.117.041384 PMC5473758 28381497

[B6] Daniel Cook. Michael H. Ralphs D. Welch B. L. (2009). Locoweed Poisoning in Livestock. Rangelands. Elsevier.

[B7] Dawn D. Patricia S. Tony H. Mike M. Barton W. Elbein A. D. (1984). Isolation and Characterization of Swainsonine from Texas Locoweed (Astragalus Emoryanus) 1. Plant Physiol. 76, 972–975. 16663983 10.1104/pp.76.4.972 PMC1064418

[B8] Dong M. Lee Y. K. Ji S. (2017). Nutlin-3 Enhances the Bortezomib Sensitivity of P53-Defective Cancer Cells by Inducing Paraptosis. Exp. Mol. Med. 49 (8), e365. 10.1038/emm.2017.112 28798402 PMC5579507

[B9] Dongjoo L. Young K. Sharmistha S. Kyeong S., (2016). Paraptosis in the Anti-cancer Arsenal of Natural Products. Pharmacol. Therap. 162, 120–133. 10.1016/j.pharmthera.2016.01.003 26802901

[B10] Dongrong Z. Chen Y. Xia L-Y. Kong J. (2019). A Purified Resin Glycoside Fraction from Pharbitidis Semen Induces Paraptosis by Activating Chloride Intracellular Channel-1 in Human Colon Cancer Cells. Int. Can. Therap. 18, 1534735418822120. 10.1177/1534735418822120 PMC7240874 30614302

[B11] Fabrizio F. Roberta M. Moretti M. Raimondi M. Marzagalli G. (2019). δ‐Tocotrienol Induces Apoptosis, Involving Endoplasmic Reticulum Stress and Autophagy, and Paraptosis in Prostate Cancer Cells. Cel. Prolife. 52, e12576. 10.1111/cpr.12576 PMC6536411 30719778

[B12] Fontana F. Raimondi M. Marzagalli M. Domizio A. D. Limonta P. (2020). The Emerging Role of Paraptosis in Tumor Cell Biology: Perspectives for Cancer Prevention and Therapy with Natural Compounds. Biochim. Biophys. Acta (Bba) - Rev. Cancer 1873, 188338. 10.1016/j.bbcan.2020.188338 31904399

[B13] Gardner D. R. Molyneux R. J. Ralphs M. H. (2001). Analysis of Swainsonine: Extraction Methods, Detection, and Measurement in Populations of Locoweeds ( Oxytropis spp.). J. Agric. Food Chem. 49, 4573–4580. 10.1021/jf010596p 11599990

[B14] Haraguchi M. Gorniak S. L. Ikeda K. Minami Y. Asano N. (2003). Alkaloidal Components in the Poisonous Plant, Ipomoea Carnea (Convolvulaceae). J. Agric. Food Chem. 51, 4995–5000. 10.1021/jf0341722 12903959

[B15] He C. Klionsky D. J. (2009). Regulation Mechanisms and Signaling Pathways of Autophagy. Annu. Rev. Genet. 43, 67–93. 10.1146/annurev-genet-102808-114910 19653858 PMC2831538

[B16] Kolb P. S. Ayaub E. A. Zhou W. Yum V. Dickhout J. G. Ask K. (2015). The Therapeutic Effects of 4-phenylbutyric Acid in Maintaining Proteostasis. Int. J. Biochem. Cel Biol. 61, 45–52. 10.1016/j.biocel.2015.01.015 25660369

[B17] Lee D. Kim I. Y. Saha S. Choi K. S. (2016). Paraptosis in the Anti-cancer Arsenal of Natural Products. Pharmacol. ? Ther., 120–133. 10.1016/j.pharmthera.2016.01.003 26802901

[B18] Li W. Huang Y. Zhao X. Zhang W. Dong F. Du Q. (2015). Swainsonine Induces Caprine Luteal Cells Apoptosis via Mitochondrial‐Mediated Caspase‐Dependent Pathway. J. Biochem. Mol. Toxicol. 28, 456–464. 10.1002/jbt.21585 24977789

[B19] Li X. Q. Ren J. Wang Y. Su J. Y. Li J. (2020). Synergistic Killing Effect of Paclitaxel and Honokiol in Non-small Cell Lung Cancer Cells through Paraptosis Induction. Cell Oncol. 44, 135–150. 10.1007/s13402-020-00557-x PMC12980790 32936421

[B20] Liu G. Wang Z. K. Wang Z. Y. Yang D. B. Liu Z. P. Wang L. (2016). Mitochondrial Permeability Transition and its Regulatory Components Are Implicated in Apoptosis of Primary Cultures of Rat Proximal Tubular Cells Exposed to lead. Arch. Toxicol. 90 (5), 1193–1209. 10.1007/s00204-015-1547-0 26082307

[B21] Lu H. Cao D. D. Ma F. Wang S. S. Yang X. W. Wang W. L. (2014). Characterisation of Locoweeds and Their Effect on Livestock Production in the Western Rangelands of China: a Review. Rangeland J. 36, 121. 10.1071/rj13105

[B22] Lu H. Ma F. Zhang L. Wang J. Wu C. Zhao B. (2015). Swainsonine-induced Apoptosis Pathway in Cerebral Cortical Neurons. Res. Vet. Sci. 102, 34–37. 10.1016/j.rvsc.2015.07.005 26412516

[B23] Lu H. Wang S. S. Zhao B. Y. (2012). Isolation and Identification of Swainsonine from Oxytropis Glabra and its Pathological Lesions to SD Rats. Asian J. Anim. Vet. Adv. 7, 822–831. 10.3923/ajava.2012.822.831

[B24] Mimnaugh E., G. (2006). Endoplasmic Reticulum Vacuolization and Valosin-Containing Protein Relocalization Result from Simultaneous Hsp90 Inhibition by Geldanamycin and Proteasome Inhibition by Velcade. Mol. Cancer Res. 4, 667–681. 10.1158/1541-7786.MCR-06-0019 16966435

[B25] Mnich K. Maurel M. Mcgrath P. Samali A. Chevet E. (2015). Controlling the Unfolded Protein Response-Mediated Life and Death Decisions in Cancer. Semin. Cancer Biol. 33, 57–66. 10.1016/j.semcancer.2015.03.003 25814342

[B26] Nawrocki S. T. (2005). Bortezomib Inhibits PKR-like Endoplasmic Reticulum (ER) Kinase and Induces Apoptosis via ER Stress in Human Pancreatic Cancer Cells. Cancer Res. 65, 11510–11519. 10.1158/0008-5472.CAN-05-2394 16357160

[B27] Nedungadi D. Binoy A. Vinod V. Vanuopadath M. Mishra N. (2021). Ginger Extract Activates Caspase Independent Paraptosis in Cancer Cells via ER Stress, Mitochondrial Dysfunction, AIF Translocation and DNA Damage. Nutr. Cancer 73 (1), 147–159. 10.1080/01635581.2019.1685113 31690139

[B28] Pfister J. A. Cook D. Panter K. E. Welch K. D. James L. F. (2016). USDA-ARS Poisonous Plant Research Laboratory: History and Current Research on Western North American Rangelands. Rangelands 38, 241–249. 10.1016/j.rala.2016.08.008

[B29] Rudolfs K. Z. Lawrence H. L. (1996). Methods in Renal Toxicology. Boca Raton: CRC Press.

[B30] Schubert U. Antón L. C. Gibbs J. Norbury C. C. Bennink J. R. (2000). Rapid Degradation of a Large Fraction of Newly Synthesized Proteins by Proteasomes. Nature 404, 770–774. 10.1038/35008096 10783891

[B31] Seo M. J. Lee D. M. Kim I. Y. Lee D. Choi M. K. Lee J. Y. (2019). Gambogic Acid Triggers Vacuolization-Associated Cell Death in Cancer Cells via Disruption of Thiol Proteostasis. Cel Death Dis. 10 (3), 187. 10.1038/s41419-019-1360-4 PMC6385239 30796201

[B32] Sperandio S. De B. I. Bredesen D. E. (2000). An Alternative, Nonapoptotic Form of Programmed Cell Death. Proc. Natl. Acad. Sci. 97, 14376–14381. 10.1073/pnas.97.26.14376 11121041 PMC18926

[B33] Sperandio S. Poksay K. Belle I. D. Lafuente M. J. Bredesen D. E. (2004). Paraptosis: Mediation by MAP Kinases and Inhibition by AIP-1/Alix. Cel Death Differ. 11, 1066–1075. 10.1038/sj.cdd.4401465 15195070

[B34] Tabas I. Ron D. (2011). Integrating the Mechanisms of Apoptosis Induced by Endoplasmic Reticulum Stress. Nat. Cel Biol. 13, 184–190. 10.1038/ncb0311-184 PMC3107571 21364565

[B35] Tang J. Guo Y. S. Zhang Y. Yu X. L. Li L. Huang W. (2012). CD147 Induces UPR to Inhibit Apoptosis and Chemosensitivity by Increasing the Transcription of Bip in Hepatocellular Carcinoma. Cel Death Differ. 19, 1779–1790. 10.1038/cdd.2012.60 PMC3469060 22595757

[B36] Wang M. Kaufman R. J. (2014). The Impact of the Endoplasmic Reticulum Protein-Folding Environment on Cancer Development. Nat. Rev. Cancer 14, 581–597. 10.1038/nrc3800 25145482

[B37] Wang S. Wang J. Yang L. Guo R. Lu H. (2019). Swainsonine Induces Autophagy via PI3K/AKT/mTOR Signaling Pathway to Injure the Renal Tubular Epithelial Cells. Biochimie 165, 131–140. 10.1016/j.biochi.2019.07.018 31356846

[B38] Wang W. B. Feng L. X. Yue Q. X. Wu W. Y. Guan S. H. Jiang B. H. (2012). Paraptosis Accompanied by Autophagy and Apoptosis Was Induced by Celastrol, a Natural Compound with Influence on Proteasome, ER Stress and Hsp90. J. Cell Physiol. 227, 2196–2206. 10.1002/jcp.22956 21866552

[B39] Wei H. Zhuang Y. Fu L. Guo B. Guo B. (2015). De Novo Transcriptome Assembly of a Chinese Locoweed (Oxytropis Ochrocephala) Species Provides Insights into Genes Associated with Drought, Salinity, and Cold Tolerance. Front. Plant Sci. 6, 1086. 10.3389/fpls.2015.01086 26697040 PMC4667070

[B40] Yoon M. J. Kang Y. J. Lee J. A. Kim I. Y. Kim M. A. Lee Y. S. (2014a). Stronger Proteasomal Inhibition and Higher CHOP Induction Are Responsible for More Effective Induction of Paraptosis by Dimethoxycurcumin Than Curcumin. Cel Death Dis. 5, e1112. 10.1038/cddis.2014.85 PMC3973237 24625971

[B41] Yoon M. J. Kim E. H. Kwon T. K. Park S. A. Choi K. S. (2012). Simultaneous Mitochondrial Ca2+ Overload and Proteasomal Inhibition Are Responsible for the Induction of Paraptosis in Malignant Breast Cancer Cells. Cancer Lett. 324, 197–209. 10.1016/j.canlet.2012.05.018 22634500

[B42] Yoon M. J. Kim E. H. Lim J. H. Kwon T. K. Choi K. S. (2010). Superoxide Anion and Proteasomal Dysfunction Contribute to Curcumin-Induced Paraptosis of Malignant Breast Cancer Cells. Free Radic. Biol Med. 48, 713–726. 10.1016/j.freeradbiomed.2009.12.016 20036734

[B43] Yoon M. J. Lee A. R. Jeong S. A. Kim Y. S. Choi K. S. (2014b). Release of Ca2+ from the Endoplasmic Reticulum and its Subsequent Influx into Mitochondria Trigger Celastrol-Induced Paraptosis in Cancer Cells. Oncotarget 5, 6816–6831. 10.18632/oncotarget.2256 25149175 PMC4196165

[B44] Zhang H. Nakajima S. Kato H. Gu L. Yoshitomi T. Nagai K. (2013). Selective, Potent Blockade of the IRE1 and ATF6 Pathways by 4-phenylbutyric Acid Analogues. Br. J. Pharmcol. 170, 822–834. 10.1111/bph.12306 PMC3799596 23869584

[B45] Zhang S. R. Zhang X. C. Liang J. F. Fang H. M. Huang H. X. Zhao Y. Y. (2020). Chalcomoracin Inhibits Cell Proliferation and Increases Sensitivity to Radiotherapy in Human Non-small Cell Lung Cancer Cells via Inducing Endoplasmic Reticulum Stress-Mediated Paraptosis. Acta Pharmacologica Sinica 41 (6), 825–834. 10.1038/s41401-019-0351-4 32066885 PMC7470873

[B46] Zhao H. Xu X. Lei S. Shao D. Huang Q. (2018). Iturin Aike Lipopeptides from Bacillus Subtilis Trigger Apoptosis, Paraptosis, and Autophagy in Cacocells. J. Cell Physiol. 234, 6414–6427. 10.1002/jcp.27377 30238995

[B47] Zheng X. Wang S. Chen D. Yang X. (2018). Swainsonine Induces Apoptosis of Rat Cardiomyocytes via Mitochondriamediated Pathway. Cell Mol. Biol. 64, 136–141. 10.14715/cmb/2018.64.5.23 29729707

