# Supplementary material for: Swainsonine Triggers Paraptosis via ER Stress and MAPK Signaling Pathway in Rat Primary Renal Tubular Epithelial Cells
Source: Front Pharmacol. 2021 Aug 10;12:715285. doi: 10.3389/fphar.2021.715285 (PMC8383073; doi:10.3389/fphar.2021.715285)
Supplement: Supplementary file 1 [file Table1.DOCX]

**Legends for supplementary figures**


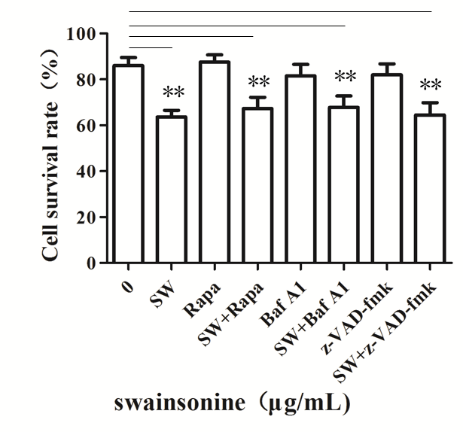

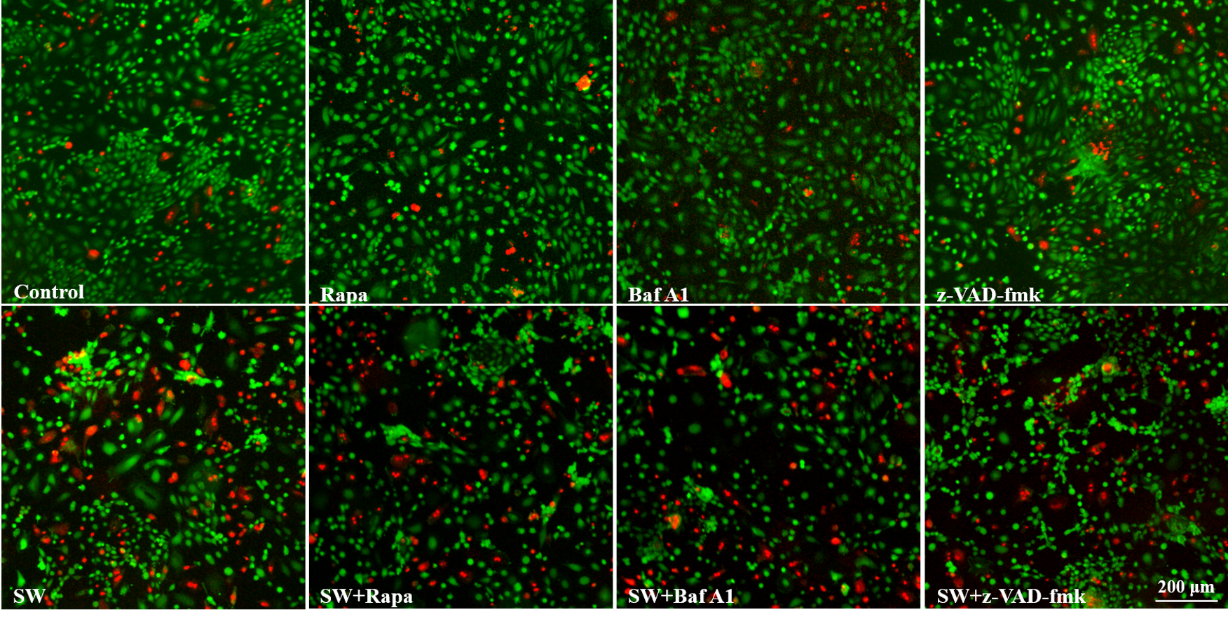


**Supplementary Figure 1.** RTECs treated with SW (400 μg/mL) in the presence of autophagic activators like Rapa, autophagic inhibitors like Baf A1, and apoptosis inhibitors like z-VAD-fmk for 24 h and cellular viability was measured using Live & Dead^TM^ Viability/Cytotoxicity Assay Kit. Analyze the number of living (green spots) and dead cells (red spots) in 3 different areas of control and SW-treated cells and the ratios were calculated.


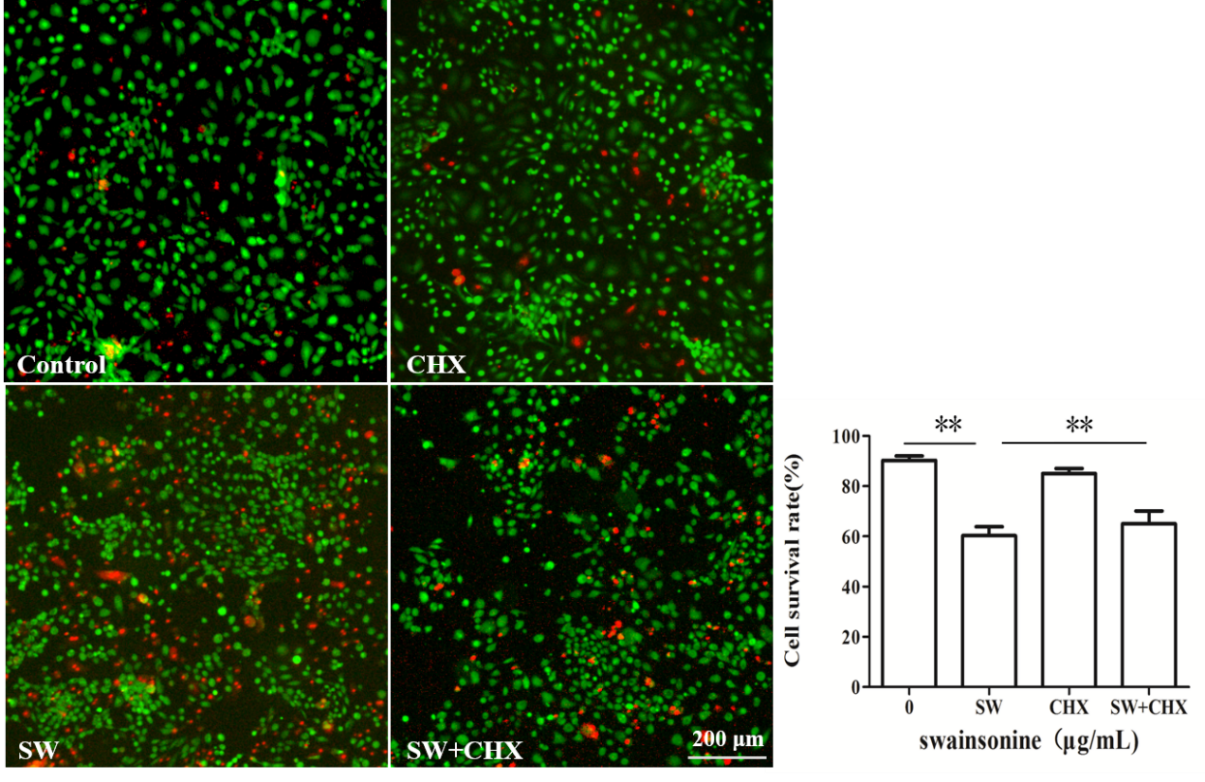


**Supplementary Figure 2.** RTECs were pretreated with CHX for 4 h and further treated with 400 μg/mL SW for 24 h and cellular viability was measured using Live & Dead^TM^ Viability/Cytotoxicity Assay Kit. Analyze the number of living (green spots) and dead cells (red spots) in 3 different areas of control and SW-treated cells and the ratios were calculated.


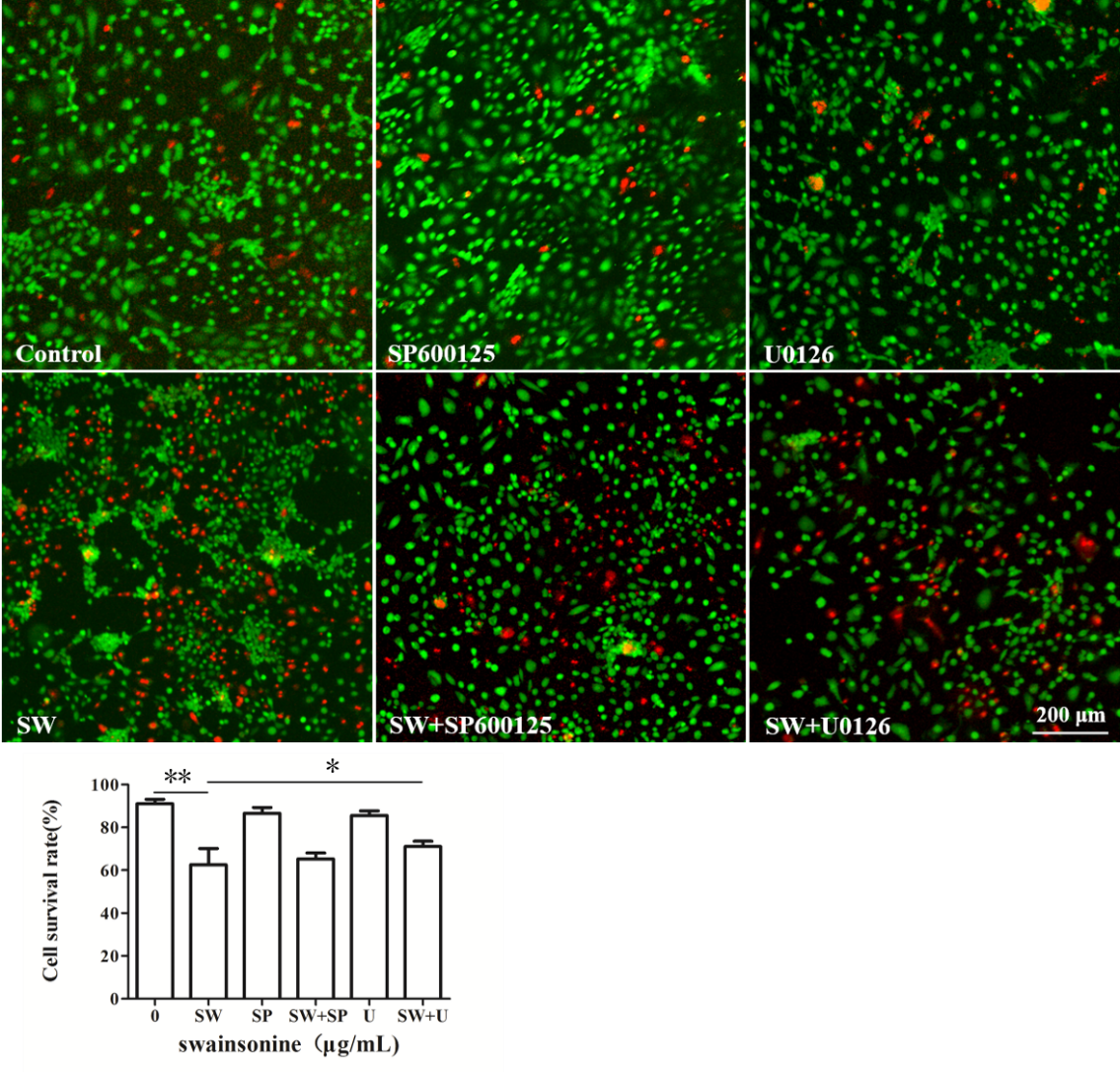


**Supplementary Figure 3.** RTECs were pretreated with SP600125 or U0126 at the indicated concentrations for 4 h and further treated with 400 μg/mL SW for 24 h and cellular viability was measured using Live & Dead^TM^ Viability/Cytotoxicity Assay Kit. Analyze the number of living (green spots) and dead cells (red spots) in 3 different areas of control and SW-treated cells and the ratios were calculated.
